# The role of inhibition in action observation treatment

**DOI:** 10.1111/j.1469-8749.2012.04356.x

**Published:** 2012-07-05

**Authors:** Alexander Kraskov

**Affiliations:** Sobell Department of Motor Neuroscience and Movement Disorders, UCL Institute of NeurologyLondon, UK

After the discovery of mirror neurons in macaque monkeys and the demonstration by a number of non-invasive studies of a similar mirror neuron system in the human brain, several researchers came up with an idea of action observation as a functional rehabilitation tool. This idea is very simple and based on a defining property of the mirror neurons: mirror neurons are active both during action execution and action observation. In other words this means that neurons involved in movement can be activated by pure observation of actions. Probably due to the simplicity of the idea it has been restated many times in different contexts, most often in context of stroke rehabilitation; but unfortunately it still remains just a very attractive hypothesis which has been confirmed only by pilot studies.^1^,^2^

Buccino et al.^3^ report another pilot study of action observation treatment (AOT) in children with cerebral palsy. Although the study was conducted on test and control groups containing only small numbers of children (8 and 7 respectively), improvement measured on the Melbourne Assessment Scale was shown for each participant in the test group. This pilot study certainly deserves to be extended into a bigger randomized clinical trial. Going beyond the pilot studies of AOT into a larger clinical trial is important for many reasons. One of them is the understanding of which patient groups can benefit from this therapy. AOT is very attractive not only because it is very easy for patients but also because the apparatus, which is essentially just video clips, can be easily adapted. The problem is that currently we do not know how the treatment should be adapted to make it more effective. Fundamentally, the ability to improve AOT resides on understanding of the cortical mechanisms which lead to improvement in patients’ behaviour. Practically, to be able to adjust AOT for individual patients, more research has to be done to reveal correlations between specific improvements achieved by patients as a result of AOT and observed actions. Another very important reason which has not been investigated and can be addressed in longitudinal trial is for how long any improvement persists. Although large clinical trials are an essential step, they by themselves do not guarantee a translation of any treatment into everyday practice. Indeed, it has been enormously difficult so far to transfer any promising advances in neuroscience into everyday clinics.^4^

One of the potential problems of the AOT hypothesis is that it assumes that the activity during action observation is similar enough to the activity during action execution. But the fact that some of the same neurons are active during these two processes does not necessarily mean that the network activity is the same. An important network difference in the brain activity during action observation is continuous inhibition of covert movements. In everyday life, observers almost never actively mirror movements of the people they observe which implies that the activity produced by the mirror neuron system in response to action observation has to be actively and continuously inhibited to prevent generation of covert movements. The necessity of such a system became even more apparent in the light of recent findings that pyramidal tract neurons, which directly project to the spinal cord, can also act as mirror neurons (i.e. mirror activity is reaching to the spinal cord).^5^ Further evidence of inhibition present during action observation is the existence of ‘suppression mirror neurons’ which decrease their activity during action observation sometimes to zero while still being very active during action execution.^5^ The important question is which cortical or subcortical network is responsible for this inhibition and whether it facilitates AOT or, in contrast, prevents it from being more effective.

In summary, AOT is a promising therapeutic tool which deserves closer investigation on a bigger scale. It can potentially benefit not only patients but be very helpful in revealing functional properties of the action observation network and its relation to the action execution network.
